# Age and Ageing During the COVID-19 Pandemic; Challenges to Public Health and to the Health of the Public

**DOI:** 10.3389/fpubh.2021.655831

**Published:** 2021-10-27

**Authors:** A. Mark Clarfield, Tzvi Dwolatzky

**Affiliations:** ^1^Ben-Gurion University of the Negev, Beersheba, Israel; ^2^Department of Geriatrics, Faculty of Medicine, McGill University, Montreal, QC, Canada; ^3^Rambam Health Care Campus, Haifa, Israel; ^4^Ruth and Bruce Rappaport Faculty of Medicine, Technion, Israel Institute Technology, Haifa, Israel

**Keywords:** COVID-19 pandemic, age, geriatric medicine, ageism, pandemic

## Abstract

The distribution of the SARS-CoV-2 virus has reached pandemic proportions. While COVID-19 can affect anyone, it is particularly hazardous for those with “co-morbidities.” Older age is an especially strong and *independent* risk factor for hospital and ICU admission, mechanical ventilation and death. Health systems must protect persons at any age while paying particular attention to those with risk factors. However, essential freedoms must be respected and social/psychological needs met for those shielded. The example of the older population in Israel may provide interesting public health lessons. Relatively speaking, Israel is a demographically young country, with only 11.5% of its population 65 years and older as compared with the OECD average of >17%. As well, a lower proportion of older persons is in long-term institutions in Israel than in most other OECD countries. The initiation of a national program to protect older residents of nursing homes and more latterly, a successful vaccine program has resulted in relatively low rates of serious COVID-19 related disease and mortality in Israel. However, the global situation remains unstable and the older population remains at risk. The rollout of efficacious vaccines is in progress but it will probably take years to cover the world's population, especially those living in low- and middle-income countries. Every effort must be made not to leave these poorer countries behind. Marrying the principles of public health (care of the population) with those of geriatric medicine (care of the older individual) offers the best way forward.

## Highlights

- The COVID-19 pandemic is a major public health challenge, with important ramifications for older persons across the globe.
- Following an approach of “*First mitigation, then (hopefully) eradication”* this Perspective article will examine the stages of coping with the pandemic, from protecting the older vulnerable population to active vaccination programs.
- These efforts raise important dilemmas, such as those relating to ageist attitudes and ethical considerations, and these will be highlighted.

“*Older people are simply our future selves”* (1)“*Erik Olsen in: Global Health and Global Aging 2007, p 352”*

## Introduction

The SARS-CoV-2 pandemic continues to affect every corner of the earth, sparing no age group. While COVID-19 can cause serious disease, hospitalization and death at any age, it largely passes over younger people and concentrates primarily in those older than 55 years of age with a logarithmic rise as age increases. Although there has been good progress in the distribution of vaccines in many wealthy countries, global efforts in achieving high levels of vaccination are slow and it remains unlikely that herd immunity will be reached. In the foreseeable future, along with an accelerated vaccine program other public health measures still need to continue. Furthermore, while wealthy countries have pushed to the front of the vaccination queue, the ability of lower income countries to purchase and provide vaccines to a significant proportion of their population remains very limited. It is reasonable to predict that it will take months or even years before COVID-19 is a thing of the past.

COVID-19 and influenza are similar yet different (2). Both are respiratory viral diseases caused primarily by the spread of droplets, aerosols and to a lesser degree by fomites. Both can kill, usually via the development of a viral pneumonia exacerbated by systematic complications. Both viruses threaten anyone with co-morbid risk factors and this hazard rises logarithmically with age. Like influenza, the spread of COVID-19 seems at least to be mitigated but not eradicated by the public health triad of physical (not “social”) distancing, strict use of facemasks and frequent hand-washing. Although most infected with SARS-CoV-2 survive, the infection-mortality rate and the case-fatality rate are approximately 10 times higher than that for influenza (2).

Risk factors for both diseases are similar. Immunosuppression and comorbidities (particularly hypertension, heart and lung disease, diabetes mellitus and obesity) offer higher rates of complication and death from both diseases. While healthy aging is to be encouraged, increased chronological age is still a clear and independent risk factor for poor outcomes (3, 4).

It must be emphasized that when referring to the group of “older persons” we are relating to a heterogeneous group. This variability exists within each age sub-cohort, reflecting varying levels of socio-economic status, and the presence of comorbidity, frailty and cognitive impairment. Generally, there is a huge difference in risk between those older persons living in long-term care nursing homes and those dwelling at home, a majority in every country. This paper will deal only with the latter. An excellent approach to the care of older institutionalized persons can be found in a recent review (5).

In this article we will relate to the stages of coping with the COVID-19 pandemic from a public health perspective with an emphasis on the older population. We will follow the path of*: “First mitigation, then (hopefully) eradication.”*

## First Mitigation

### Protecting Those Who Are Vulnerable

Risk factors matter at *every* age and those at increased risk should make vigorous efforts to protect themselves from the virus. As mentioned previously chronological age is an independent risk factor constituting a continuous variable (3, 4). An astounding fact is that if infected with the coronavirus, the “old-old” (>85 years) have at least 1,000 times the risk of dying than does a child aged 0–4 years (6). Male gender and low socio-economic status are also risk factors.

Initially, most authorities did not succeed in adequately informing and warning at-risk groups (including older persons) of the dangers of COVID-19 infection. Subsequently, clear, evidence-based guidelines were published, such as those found in United States CDC website (6). In the interest of Public Health and the health of the public, all relevant governmental departments should strive to distribute such material in all pertinent languages—in print, radio, television and via social messaging. The most effective epidemiological tool is still physical distancing. However, this practice constitutes a double-edged sword especially for older persons for whom it can result in severe psychological and social side-effects (7). With careful planning these side-effects can be at least partially mitigated. And we have also learned that many older persons are actually quite resilient (8).

These protective efforts must be carefully coordinated among health and social services, volunteer groups and local authorities as well as with appropriate support from central government—sadly not always easy to accomplish (9). For Israel, a detailed plan has been published elsewhere (10). For those “shielded“ at home, health and other various services need to be organized and delivered to older persons in a timely way (e.g., food and medication). In the absence of family support, many older persons will also need help with “simple” measures such as accessing the services of a plumber, electrician, handyman or telephone technician.

Although mistakenly enforced during the first wave in many places around the world, at risk and especially older persons must not remain ”locked up“ at home. Appropriately masked, they can go out to grocery stores and use essential services, such as doctor visits. There is no good reason to discourage even high-risk persons from meeting friends and/or family members outside or in a well-ventilated room, maintaining the 2-meter limit with all parties wearing masks. ”Physical“ distancing need not cause ”social“ separation more than is absolutely essential.

For their part, government and local authorities can help lower risk by organizing and enforcing special “elders' hours” at the supermarket, such as 10:00–12:00 three times per week, during which time entry is restricted only to those over age 60 and with mandatory masking. Furthermore, stores can organize ”one way“ signs so that shoppers pass through a store in an orderly manner, thus minimizing contact between shoppers. Business interests can be conscripted to help offer sensible and safe shopping practices (11).

### Influenza and COVID-19: A Potential Nightmare Scenario

Clearly, at least in wealthy countries, we are beginning to observe the positive effects of the widespread use of newly licensed and safe vaccines that are effective against COVID-19 infections. In expectation of the winter season this year there were also legitimate fears of a possible “twindemic” of influenza and COVID-19 which could well-have occurred. Luckily this year for reasons that are still not clear we got away lightly in the flu domain. But for next season an orderly and focused flu vaccine program will need to be bolstered. As pointed out in the NY Times recently, Dr. Anthony Fauci, director of the U.S. National Institute of Allergy and Infectious Diseases, ”…. has been imploring people to get the flu shot, 'so that you could at least blunt the effect of one of those two potential respiratory infections”' (12). This is good advice for next fall—and this advice should be heeded both by the individual and by health authorities.

### Triage and Advanced Directives

Older persons are probably not more likely than younger ones to be infected with SARS CoV-2 virus, yet if infected they suffer a much higher incidence of complications and death. If the number of such cases rises well-beyond the ability of health services to cope with the resultant demand for hospital/ICU beds, ventilators, and specially trained teams needed to activate them, the vexed question of triage arises (13). Many countries have been faced with this challenge, being forced to activate emergency practices and public health contingency measures in order to ensure a fair, transparent and publicly agreed-upon system. These measures should be planned in advance since triage cannot be organized during a crisis situation, as happened in northern Europe at the advent of the pandemic (14).

Although many older persons (and some younger people for that matter) may not wish to be intubated should their clinical situation deteriorate, most have not signed advanced directives to avoid such an eventuality (15). Having relevant discussions with family members and the timely signing of the requisite documents will often ensure that preferences will be followed and autonomy respected.

Furthermore, for those who do decide to eschew such treatments, not only would a vexed triage decision be avoided, but scarce resources would be conserved for those of any age. In this way distributive justice would be maximized without the need for any overt triage decisions. With respect to older patients suffering from cognitive decline, they especially would have a difficult time understanding what is wanted of them if the need for an intensive care unit is raised. As well, for those who are frail, and for those of advanced old age with comorbidity, the most humane practice might be to offer skilled palliative care in an effort to encourage non-maleficence. To act otherwise would simply lead in many cases to a “bad death” (16).

### Protecting Older People and Ageism

Much has been written about ageism, which is defined by the WHO as “the stereotyping, prejudice, and discrimination against people on the basis of their age (alone).…. For older people, ageism is an everyday challenge. Overlooked for employment, restricted from social services and stereotyped in the media, ageism marginalizes and excludes older people in their communities” (17).

The COVID-19 crisis may facilitate a recrudescence of the doleful phenomenon of ageism, for example through “locking up” older persons in order to “preserve” hospital beds for younger people and/or to “save the economy.”

In this vein, the recent use in the UK of the “Stay at Home, Save the NHS” message was confusing and possibly harmful to some, although that was clearly not the intention of those who offered it. While the NHS is a highly respected institution, some have suggested that this message could inadvertently mislead the public, especially older persons. Some people avoided coming to hospital, suffering heart attacks and strokes at home and delaying cancer chemotherapy. In contrast, people hoped that the goal of their health care system was to care for the population and not the other way around.

However, there are other issues touching on ageism which have been less widely discussed. For example, in the name of defending against ageism, some have argued that healthy older persons who are in better shape than sick younger people should not be singled out as particularly at risk. It has been posited by some social gerontologists that even to *claim* that chronological age is an independent risk factor comprises an ageist approach.

However, the epidemiological facts are clear (3). The fact that a healthy 80 year old (with no comorbidity) still has a significantly shorter life expectancy than a 65 year old person with up to five (!) comorbid conditions should put this myth to rest (18). Understandably, many older persons and some of their advocates may wish to think differently for otherwise laudable reasons. However, this misperception can lead to poor advice and faulty (and dangerous) decisions, both personal and by the relevant authorities. In our view, it is ageist and disrespectful of an older person's autonomy *not* to make these facts clear.

In a related phenomenon, the evocative term “gray on gray” ageism has been described by David Oliver who offers “What is undoubtedly ageist is a collective fear of aging and death in our societal and media values, meaning that appearing old is seen as being diminished, invisible, and unvalued by society. This in turn leads to older people themselves “othering” any older people they see as being vulnerable, different from their more youthful and active selves. This can lead to “gray on gray” ageism” (19).

### Public Health Systems and Older Persons

Well-before the COVID-19 challenge, it was clear that the whole population, and especially older persons, require strong primary care backed up by excellent and adequate hospital services. The pandemic has made this truism abundantly clear. However, even in those countries with well-organized and generously funded medical systems, such as those for example in the UK, northern Italy and a significant number of American states, many jurisdictions have struggled to maintain equilibrium. Some have claimed that market driven distortions in the structure of health systems over the past decades have left otherwise well-funded and previously well-regarded medical jurisdictions woefully unprepared for the pandemic (20).

During such a crisis, careful thought must be given as to how best to support these systems and, especially when this crisis ends, how to prepare ourselves for the next pandemic. The shocking fact is that many countries (for example the United States, among others) actually had detailed pandemic plans at the ready, but when the virus rolled in these plans were not implemented. This counterintuitive and destructive phenomenon should give us pause to reflect as to how and why this happened (21) and hopefully to re-structure public health systems accordingly.

## Then (Hopefully) Eradication

### In the End, a Vaccine

Several vaccines are now authorized for emergency use (and more than 150 vaccine candidates are in the pipeline). The ongoing widespread vaccination programs will hopefully safely build global herd immunity (22). This phenomenon has clearly been shown to be the case in countries such as Israel and we will likely observe similar positive effects in other countries as they successfully roll out their own vaccination programs. The need for two doses in some of the authorized vaccines, as well as the use of booster doses of the vaccine (initiated in Israel in July 2021), further complicates matters as manufacturers struggle to produce enough vaccine to cover the whole world at once, not to speak of the vexed question of payment. Given the practical issues involved with the distribution and administration of these products, especially for those requiring a hyper-cold vaccination chain, difficult public health decisions need to be made. These will need to be aligned in an analogous way with the principles of medical ethics that define the fraught triage considerations alluded to above.

Related to the order of vaccination, older people are disproportionately affected by COVID-19 (23) and should be prioritized. However, as alluded to above, this population comprises a heterogeneous group. In recognition of this fact, one prestigious American group, the National Academies of Science Engineering and Medicine has indeed recommended that older people stand toward the front of the vaccination line but that they be considered the second of two sub-groups (24). Just after essential workers (including health care personnel) would be older persons residing in long-term nursing institutions, given their extremely high risk. These older persons should be followed by community-dwelling, presumably healthier older people. Broadly speaking this makes sense, but it must be kept in mind that there is a significant group of very frail older persons at home looked after by devoted family members who may actually be at higher risk than some of those in nursing homes. The devil will be in the details.

## Some Final Thoughts

### Other Ethical Issues

Most of the public health issues dealt with in this paper have complex ethical dimensions, some of which have been addressed above, at least in part. Public health practice, as is the case in clinical medicine, must be supported via ethically sound policies and maneuvers (10, 25, 26). But in brief, the principle of supporting autonomy connects with the necessity of providing accurate information with respect to age as a risk factor as well as facilitating the use of advanced directives. Supporting non-maleficence brings to mind efforts to protect the demented and very frail from spiraling into a “bad death“ via inappropriately aggressive and futile therapy (16). Beneficence has been adduced to help older persons shelter at home with maximum social support and minimal suffering as well as being offered priority for receiving the new vaccines when they become available. Finally, the principle of distributive justice is relevant to triage for hospital services and the order of vaccine distribution. The pandemic is a moving target, both geographically and temporally. At the time of writing, there is a rapid rise in the number of new cases infected by the delta variant of COVID-19. While the delta variant is associated with a higher rate of hospital admissions than the alpha variant, this rise in admissions is seen more in younger persons (27). Since vaccinations seem to be protective against the delta variant, this probably reflects the lower rate of vaccinations in younger people compared to the older population. As this third wave arises in many countries, constant vigilance is essential in order to allow for the proper design of rational and humane public health policies.

### High Number of Older Persons in Low Income Countries

Many consider the issue of aging to be restricted to high income countries (HICs); this perception is an error. Although the relative number of older persons is still higher in HICs than that observed in lower and mid-income countries (LMICs), in absolute terms more older persons today reside in the latter than the former (28). As well, due to more difficult environmental conditions in which these older people have lived their lives, many are ”sicker earlier" than their respective cohorts in wealthier countries (29, 30). Furthermore, these older persons are beginning to suffer from the same non-communicable diseases as do their counterparts in HICs but in LMICs these maladies are usually grossly under-diagnosed and treated. As such, older persons with co-morbidity are actually quite numerous in LMICs.

Furthermore, the general socioeconomic damage wrought by the pandemic, especially in vulnerable LMICs, is causing a rapid rise in poverty with resultant food insecurity which threatens young and old alike, rendering them all more susceptible to the effects of virus—both direct and indirect (31). At least in theory, these conditions will render elders in LMICs susceptible to COVID-19, although this vulnerability may at least in part be mitigated by the younger age structure of the population. The truth is that because of underreporting and less than robust vital statistics infrastructure, we do not really have an accurate picture. But the signs are clearly not promising and need further attention.

Finally, with respect to the global availability of vaccines, the HICs are grabbing the first batches, leaving those poorer countries which cannot compete to wait at the end of the line. This scenario is not only unjust, it is probably in the interest of all, both rich and poor countries, to design an equitable system where people wherever they live are prioritized according to the criteria mentioned above. The international vaccine alliance GAVI makes the point eloquently in a recent position statement (32) and the COVAX initiative which it is co-sponsoring is making strenuous efforts to encourage a more equitable distribution of vaccines around the world (33).

## Conclusion

The COVID-19 pandemic is the most serious public health crisis to challenge the globe in a century. However, from the scientific and healthcare point of view, at least in the HICs, today's health systems are in much better shape than they were during the flu epidemic of 1918–1920. That being said, our world has developed and aged and the behavior of the SARS-CoV-2 virus is not yet fully understood. During the pandemic of a century ago, paradoxically, most complications and death occurred in young healthy persons (average age of death 28!) Today, while COVID-19 mostly spares younger people from severe comorbidity, at the other end of the spectrum older persons offer an extremely vulnerable target. As well, at all ages we can now save many more lives with the tools of modern medicine. It is well to remember that in 1918, although oxygen therapy was beginning to be understood, it did not become widely used by physicians until many decades later (34).

Furthermore, the social and economic pressures wrought by the virus and the subsequent efforts to mitigate its effect are causing unprecedented socio- economic damage and strain across the globe—in rich and poor countries alike. There are also some intimations of inter-generational conflict as well as a possible erosion in political and human rights; this along with a recrudescence of totalitarian political practices in many countries such as attacks on the media and judiciary, exacerbated by the strains induced by the pandemic (35).

The challenges SARS-CoV-2 present to public health and the health of the public will require all of our ingenuity, both medical and political, in order to mitigate the damage wrought by the pandemic until effective vaccines become widely available. Due to their biologically induced susceptibility to SARS-CoV-2 and the attendant social vulnerabilities induced by the pandemic and our efforts to curb it, many older persons around the globe will require special considerations and protections.

## Data Availability Statement

The original contributions presented in the study are included in the article/supplementary material, further inquiries can be directed to the corresponding author/s.

## Author Contributions

AC conceptualized and wrote the manuscript. TD edited, revised, and added to the manuscript. Both authors contributed to the article and approved the submitted version.

## Conflict of Interest

The authors declare that the research was conducted in the absence of any commercial or financial relationships that could be construed as a potential conflict of interest. The reviewer MO declared a past collaboration with one of the authors TD to the handling editor.

## Publisher's Note

All claims expressed in this article are solely those of the authors and do not necessarily represent those of their affiliated organizations, or those of the publisher, the editors and the reviewers. Any product that may be evaluated in this article, or claim that may be made by its manufacturer, is not guaranteed or endorsed by the publisher.
